# Global trends and hotspots on childhood obstructive sleep apnea: a 10-year bibliometric analysis

**DOI:** 10.3389/fped.2023.1160396

**Published:** 2023-05-10

**Authors:** Chao Wang, Kai Sun, Siyao Zhang, Huiying Hu, Yuanyuan Lu, Kai Liu, Zhenkun Yu

**Affiliations:** ^1^ School of Medicine, Southeast University, Nanjing, China; ^2^ Department of Otolaryngology Head and Neck Surgery, BenQ Medical Center, The Affiliated BenQ Hospital of Nanjing Medical University, Nanjing, China

**Keywords:** obstructive sleep apnea, children, global trends, hotspots, bibliometric analysis

## Abstract

**Background:**

Obstructive sleep apnea (OSA) is children's most common sleep-related breathing disorder. It may develop a wide range of severe complications if not diagnosed promptly and treated effectively. However, Childhood OSA has not specifically been analyzed using a bibliometric approach.

**Methods:**

We respectively collected the research results of childhood OSA from 2013 to 2022 through Web of Science and PubMed. Vosviewer, CiteSpace, and bibliometric online analysis platforms were used for visualizing and analyzing the literature. The MeSH terms were bi-clustered using the Bibliographic Item co-occurrence Matrix Builder (BICOMB) and graph clustering toolkit (gCLUTO) to identify the hotspots.

**Results:**

4022 publications were finally identified on childhood OSA from 2013 to 2022. The United States has the largest number of publications (1902), accounting for 47.29%. University of Cincinnati is the most productive organization (196), followed by the University of Pennsylvania (151). The most prolific journal was the International Journal of Pediatric Otorhinolaryngology, with 311 documents published. In comparison, Pediatrics is the most cited journal (6936). Gozal D ranked highest among all authors in publication (192). Burst detection shows continuous positive airway pressure, Robin sequence, and nocturnal oximetry are recent keywords of great interest to researchers. Five hotspots were identified by co-word biclustering.

**Conclusion:**

Research over the past ten years has been fruitful, establishing the foundation for childhood OSA. Clusters (0-4) of high-frequency Major Mesh topics have attracted extensive attention. Evaluation and treatment methods of childhood OSA remain major focuses. We believe this article will provide other researchers with new directions and may contribute to a future breakthrough in this field.

## 1. Introduction

Obstructive sleep apnea (OSA) is the most common sleep-related breathing disorder in children and is characterized by repetitive partial or complete collapse of the upper airways during sleep (1). In the past decade, it has undoubtedly received increasing attention around the world. Children with obstructive sleep apnea may develop a wide range of severe complications if not diagnosed promptly and treated effectively, including abnormal maxillofacial development (adenoid face), behavioral problems, learning disabilities, growth retardation, neurocognitive impairment, metabolic disorders, hypertension, pulmonary hypertension, and even increased risk of cardiovascular events in adulthood (2–5). However, Pediatric OSA has never been analyzed using a bibliometric approach.

Bibliometric analysis is now the most effective tool for examining historical research trends in a specific field (6). By using qualitative and quantitative analysis, it presents the research contributions of different countries, institutions, journals, and authors in scientific fields and forecasts research trends and hotspots (7). Meanwhile, it is imperative to clarify that hotspots and frontiers refer to problems that are not yet resolved in a specific field and are highly concerned by global scholars. Also, the research directions need to be identified urgently and with great importance in the future. Overall, bibliometric analysis has been instrumental in the formulation of policy and clinical guidelines on various diseases.

In this study, we aim to comprehensively assess the global academic status and hotspots relevant to childhood OSA, and to predict future trends in this field.

## 2. Methods

### 2.1. Data sources and search strategies

A comprehensive online search of the literature on childhood OSA from 2013 to 2022 was conducted using the Science Citation Index Expanded (SCI-EXPANDED) database *via* the Web of Science (WoS) Core Collection. For the precaution of bias in database updates, all searches were completed on January 2, 2023. The search strategy was TS = (“obstructive sleep apn*”) AND ALL = (pediatri* OR child*). We set the language to English and only saved original articles and reviews. Medical Subject Headings (MeSH) terms are standardized vocabulary terms for reflecting the main thrust of a literature review. Moreover, it could be used for performing continuous biclustering analysis. Similarly, we searched PubMed based on the screening criteria of (Sleep Apnea, Obstructive[MeSH]) AND (Pediatri* OR Child*), which was developed by the National Center for Biotechnology Information (NCBI) of the National Library of Medicine (NLM).

### 2.2. Data collection and statistical analysis

Two authors (LK and SK) independently collected all data, with an agreement rate of 0.95, which implies a high level of agreement (8). After converting Web of Science data to txt format, Vosviwer version 1.6.18 (Leiden University, Leiden, The Netherlands) and CiteSpace version 6.1.R3 (Drexel University, Philadelphia, PA, USA) were used to screen and compute the data, as well as the Online Analysis Platform of Literature Metrology (9–11). The data downloaded from PubMed were imported into Bibliographic Item Cooccurrence Matrix Builder (BICOMB), which was developed by China Medical University (12). Eventually, gCLUTO version 1.0, Graphical Clustering Toolkit, was applied for biclustering analysis for MeSh major topics (13).

All literature characteristics were selected to analyze respectively. VOSviewer was applied to analyze and visualize bibliometric data, such as countries, institutions, journals, and authors. Further, we created network visualization plots and density visualization. In the meantime, CiteSpace has a special analysis method called burst-time analysis, a computational technique for identifying the time point when a certain research direction becomes a hotspot. Therefore, we generated a plot of authorship-keyword bursts from a collection of bibliographic records, which will allow us to explore historical points and potential future directions. Additionally, clustering with different numbers of clusters was redirected so that the matrix visualization would result in the most reasonable outcome. In the clusters, we illustrated the semantic relationships between MeSH major topics and the source literature using matrix and mountain visualizations.

## 3. Results

### 3.1. Analysis of annual publications

**Figure 1:**
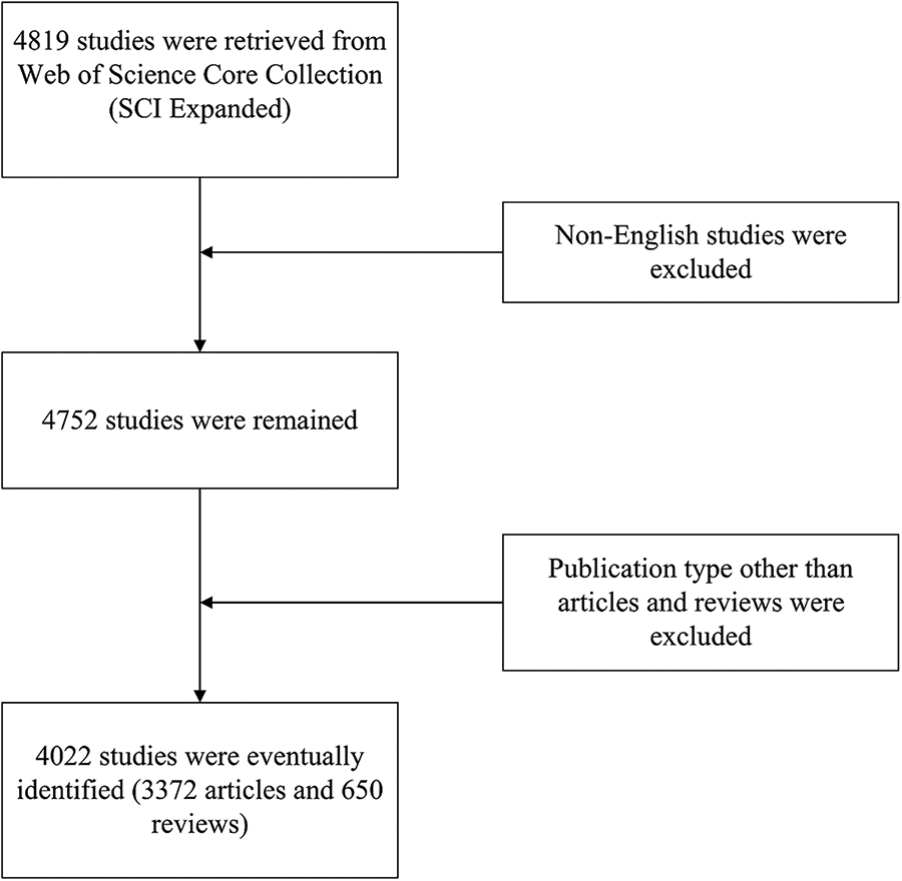
Flow chart of the inclusion process.

**Figure 2:**
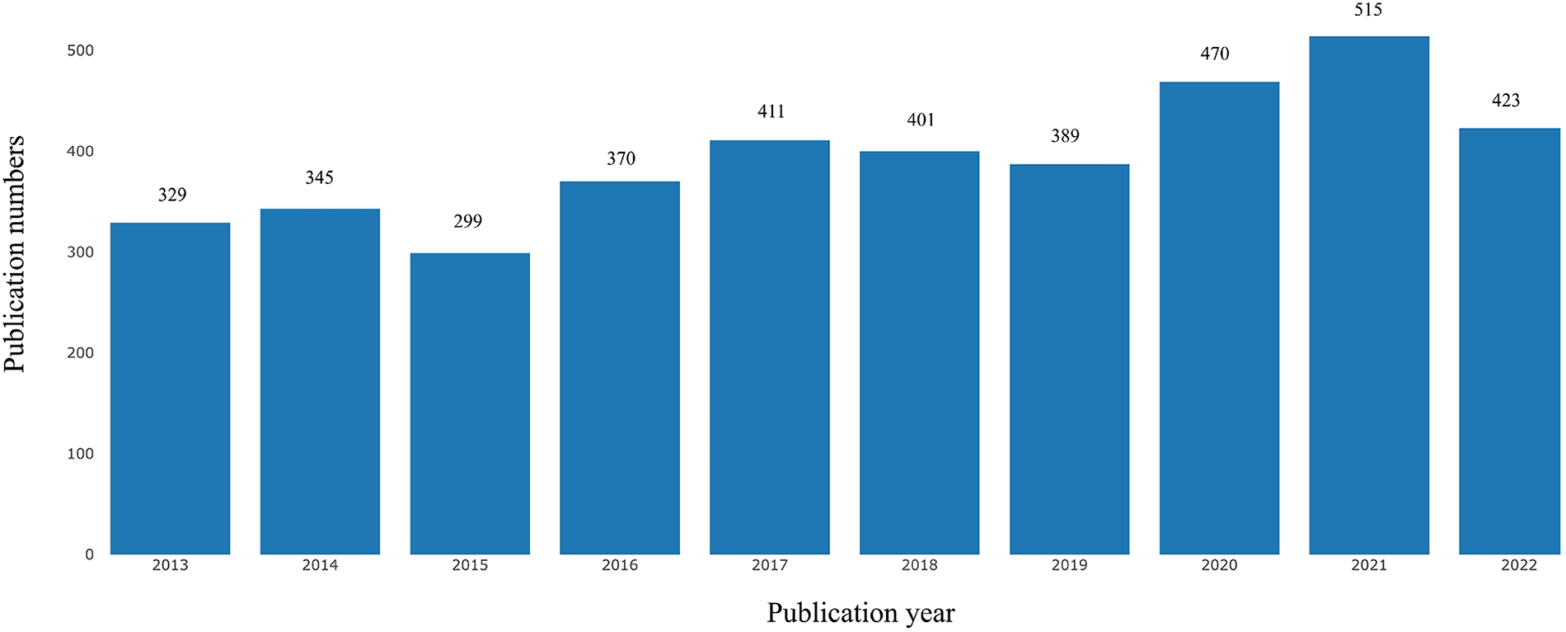
Growth trends of publications on childhood OSA from 2013 to 2022.

As shown in the flowchart (Figure 1), the initial search retrieved a total of 4,819 publications. After excluding the literature that does not belong to articles and reviews and further limiting language to English, 4,022 articles were finally identified. Publications related to Childhood OSA showed an overall increasing trend from 2013 to 2022, despite a slight decrease in 2015, 2018, 2019, and 2022 (Figure 2).

### 3.2. Performance of countries/regions

**Figure 3:**
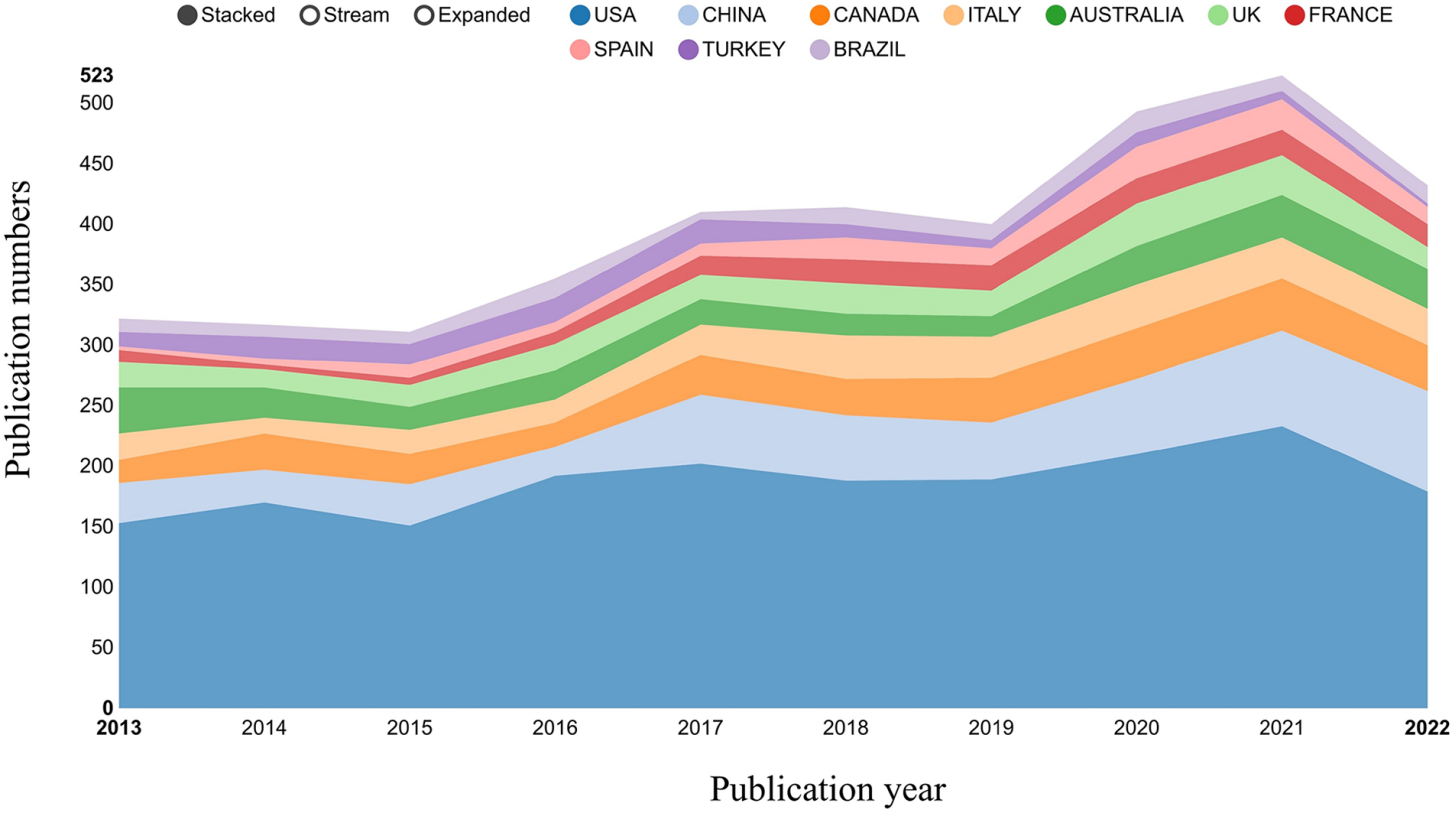
Growth trends of the top 10 countries on childhood OSA from 2013 to 2022.

**Figure 4:**
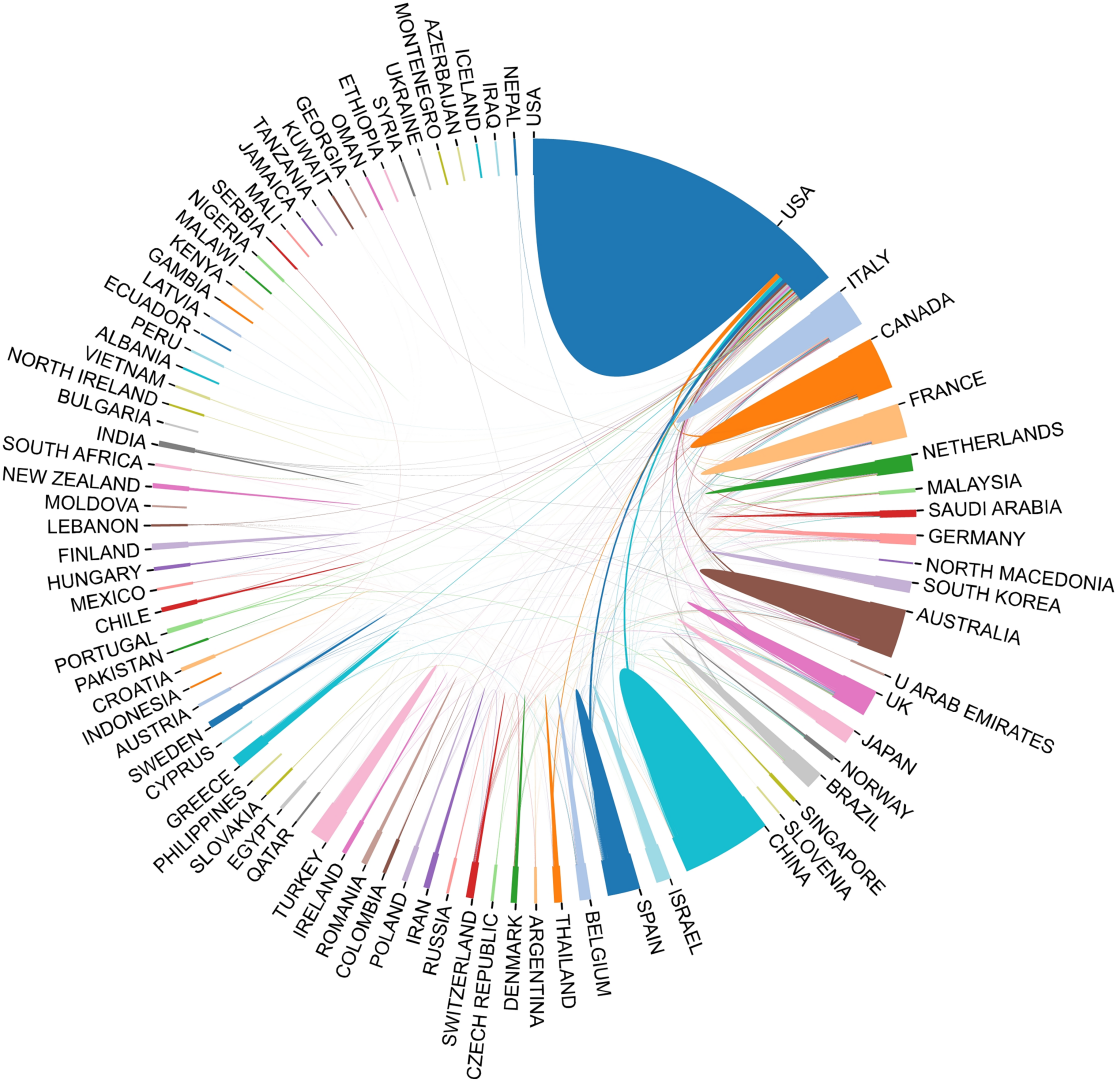
The cooperation between countries on childhood OSA. The thickness of the line between two countries indicates their cooperative relationship strength.

Over the past decade, at least 64 countries or regions have published studies on childhood OSA. The top 10 national publication is presented in Table 1 and Figure 3. The United States (1,902, 47.29%) was the largest publication contributor, followed by China (510, 12.68%), Canada (326, 8.11%), Italy (276, 6.86%), and Australia (264, 6.56%). Figure 4 demonstrates the cooperation among the countries/regions. The cooperation between the United States and Canada was the closest, followed by the cooperation between the United States and China. Furthermore, Figure 4 also indicates that there is a lack of academic exchange between countries with abundant publications and countries with a limited number of publications.

**Table 1:** Top 10 productive countries and regions.

Rank	Country	Articles	Citations	Total link strength
1	USA	1,902	45,031	906
2	China	510	6,535	185
3	Canada	326	18,310	413
4	Italy	276	6,991	383
5	Australia	264	5,330	282
6	UK	232	7,875	462
7	France	150	3,262	278
8	Spain	138	4,239	317
9	Turkey	131	1,987	134
10	Brazil	129	1,756	187

### 3.3. Performance of organization

**Figure 5:**
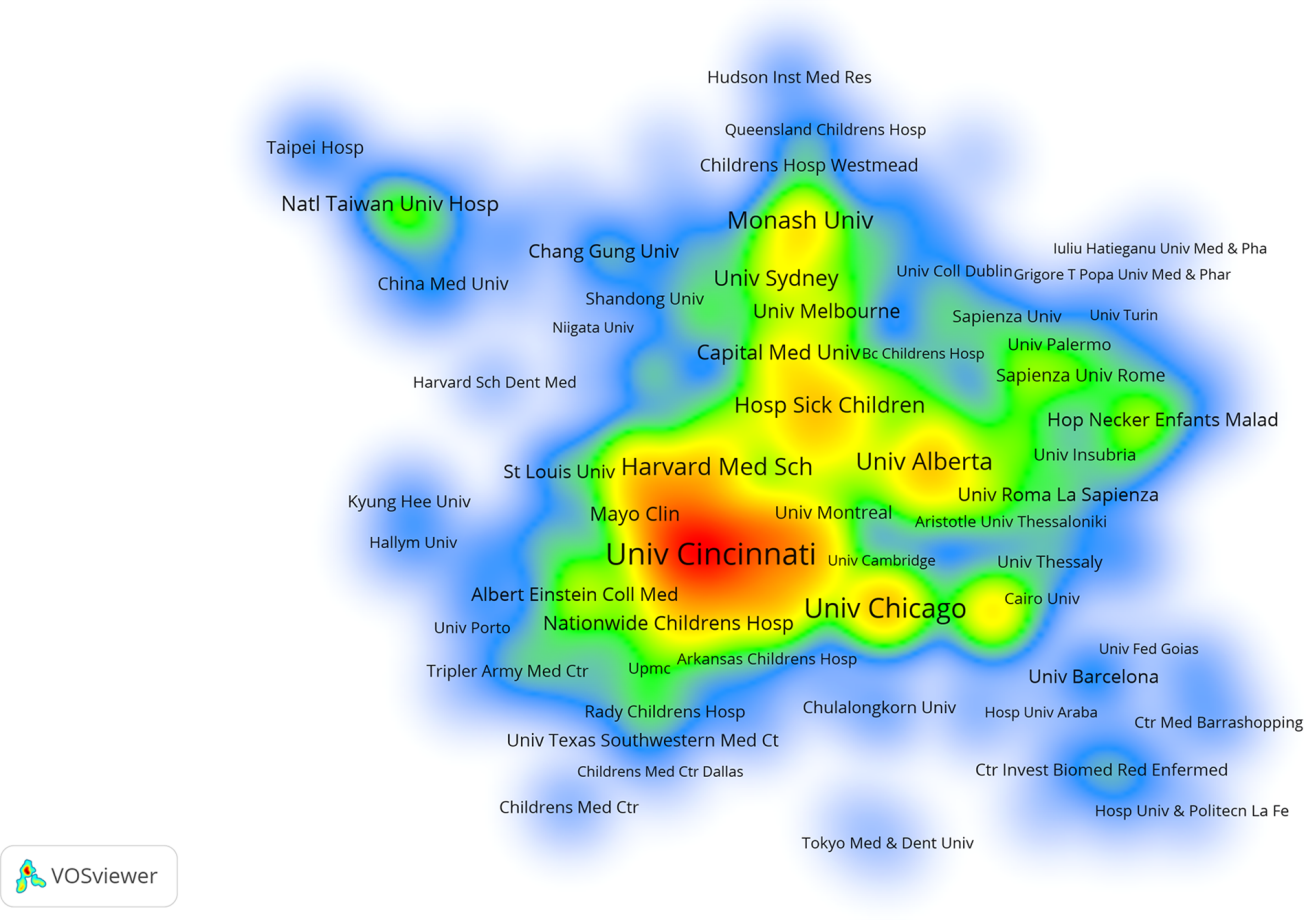
The network visualization of institutions that contributed at least 5 articles on childhood OSA. A gradual increase in the number of publications is represented by color changes from blue to green to yellow to red.

Our study showed that 3,759 organizations had published studies on childhood OSA. We list the top 10 most productive organizations in Table 2. The most prolific organization was the University of Cincinnati (196), followed by the University of Pennsylvania (151) and the University of Chicago (135). Among the top 10 institutions, 7 of 10 were from the United States, corresponding to the distribution by country and region. Further, the research density visualization was plotted by Vosviewer (Figure 5).

**Table 2:** Top 10 productive organizations.

Rank	Organization	Country	Documents	Citations
1	University of Cincinnati	United States	196	4,812
2	University of Pennsylvania	United States	151	4,322
3	University of Chicago	United States	135	6,726
4	University of Missouri (Columbia)	United States	114	1,032
5	University of Washington	United States	87	2,103
6	Monash University	Australia	83	1,593
7	University of Alberta	Canada	83	1,019
8	University of Toronto	Canada	83	1,175
9	Harvard Medical School	United States	82	1,994
10	University of Michigan	United States	82	2,294

### 3.4. Performance of journals

**Figure 6:**
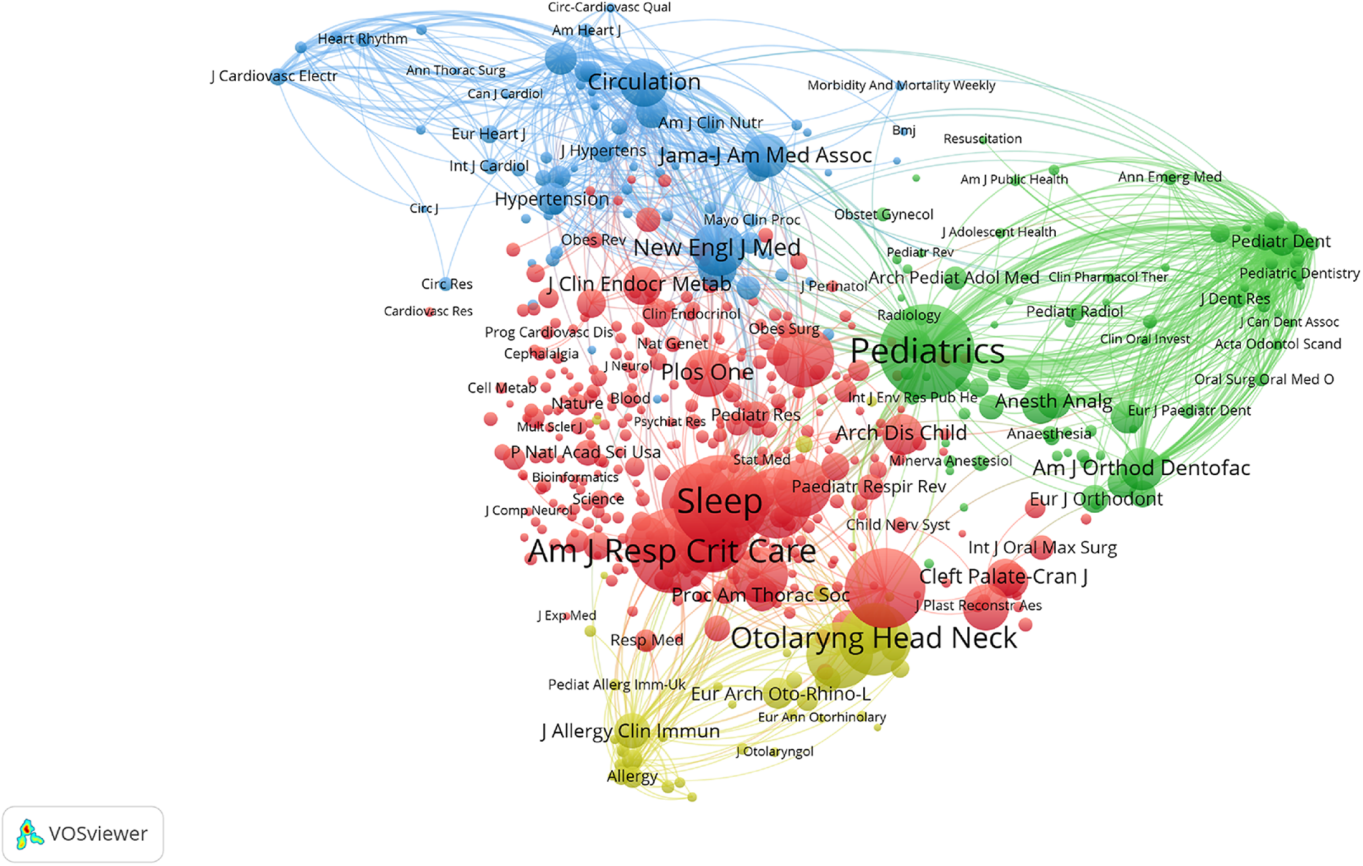
The co-citation network of journals cited at least 50 times on childhood OSA. In network visualization, colors show clustering. The red cluster mainly represents sleep-related journals; the Green cluster mainly represents pediatric journals; the Blue cluster mainly represents cardiovascular journals; the Yellow cluster mainly represents Otorhinolaryngology and Allergy journals.

There were a total of 771 journals that published articles on childhood OSA. We list the top 10 journals of publication in Table 3. The most prolific journal was the International Journal of Pediatric Otorhinolaryngology, with 311 documents published. Then it comes to Journal of Clinical Sleep Medicine, with 207 documents published. Two journals have the characteristic of IF > 5: Otolaryngology-Head and Neck Surgery (5.591) and Sleep (6.313). In addition, the top 10 journals of citation in childhood OSA research are listed in Table 4. We also conducted a co-citation analysis and plotted a network visualization (Figure 6) for journals by Vosviewer. Pediatrics is the most cited journal (6,936 citations), followed by Sleep (6,761 citations) and American Journal of Respiratory and Critical Care Medicine(5,491 citations).

**Table 3:** Top 10 journals of publication in childhood OSA research.

Rank	Journal Title	Country	Articles	Impact Factor (2021)	H-index
1	International Journal of Pediatric Otorhinolaryngology	Netherlands	311	1.626	69
2	Journal Of Clinical Sleep Medicine	United States	207	4.324	78
3	Sleep Medicine	Netherlands	196	4.842	107
4	Laryngoscope	United States	138	2.97	134
5	Sleep and Breathing	Germany	128	2.655	55
6	Otolaryngology-Head and Neck Surgery	United States	111	5.591	109
7	Sleep	United States	98	6.313	184
8	Pediatric Pulmonology	United States	93	4.09	98
9	Journal Of Craniofacial Surgery	United States	62	1.172	66
10	European Archives of Oto-Rhino-Laryngology	United States	56	3.236	61

**Table 4:** Top 10 journals of citation in childhood OSA research.

Rank	Journal Title	Country	Citations	Impact Factor (2021)	H-index
1	Pediatrics	United States	6,936	9.703	311
2	Sleep	United States	6,761	6.313	184
3	American Journal of Respiratory and Critical Care Medicine	United States	5,491	30.528	343
4	International Journal of Pediatric Otorhinolaryngology	Netherlands	4,883	1.626	69
5	Sleep Medicine	Netherlands	4,238	4.842	107
6	Chest	United States	4,105	10.262	267
7	Otolaryngology-Head and Neck Surgery	United States	4,018	5.591	109
8	Journal of Clinical Sleep Medicine	United States	3,768	4.324	78
9	Laryngoscope	United States	3,327	2.97	134
10	Journal of Pediatrics	United States	2,844	6.314	188

### 3.5. Performance of author

Based on our study, 4,022 articles were published by 15,947 authors with at least one article. Table 5 shows the top 10 most productive authors who published articles or reviews in the childhood OSA field. Gozal D ranked highest among all authors (192 articles), followed by Ishman SL (84 articles), Kheirandish-Gozal L (77 articles), and Nixon GM (60 articles).

**Table 5:** Top 10 authors of publication in childhood OSA research.

Rank	Author	Documents	Citations
1	Gozal, David	192	4,744
2	Ishman, Stacey L.	84	1,350
3	Kheirandish-Gozal, Leila	77	2,331
4	Nixon, Gillian M.	60	1,073
5	Horne, Rosemary S. C.	50	959
6	Davey, Margot J.	49	809
7	Marcus, Carole L.	43	1,330
8	Mitchell, Ron B.	42	1,096
9	Narang, Indra	40	554
10	Khalyfa, Abdelnaby	37	1,012

### 3.6. Burst detection with keywords

**Figure 7:**
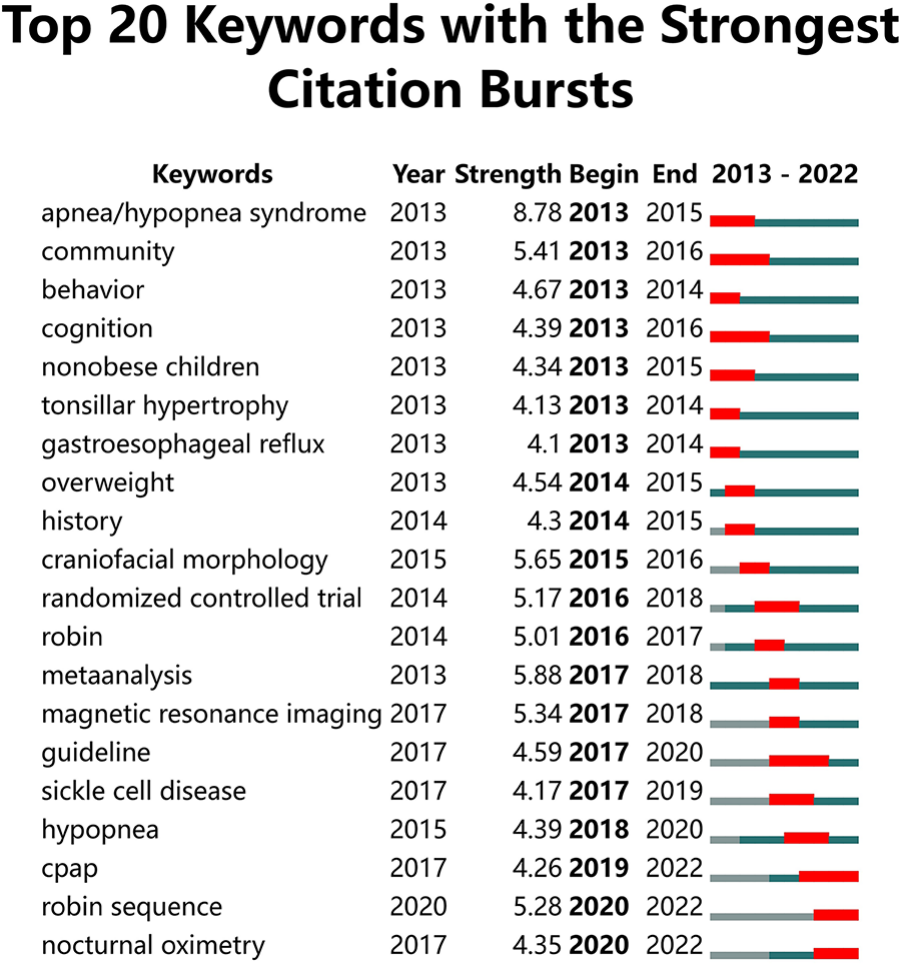
Top 20 keywords with the strongest citation burst. The green line sliced by year represents the timeline, while the red grid represents the beginning and ending year and the time interval of citation bursts.

The term “burst words” refers to keywords whose frequency of citations suddenly increases within a short period, providing insight into areas of research that are on the verge of breakthroughs (14). In Figure 7, the green line sliced by year represents the timeline, while the red grid represents the beginning and ending year and the time interval of citation bursts. Apnea/hypopnea syndrome itself ranked first, with the strongest burst strength over the past decade (8.78), followed by meta-analysis (5.88), craniofacial morphology (5.65), community (5.41), magnetic resonance imaging (5.34), and Robin sequence (5.28). From 2020 to 2022, OSA-related research has focused on continuous positive airway pressure (CPAP), Robin sequence, and nocturnal oximetry, suggesting that these topics are currently of great interest to researchers.

### 3.7. Research hotspots based on MeSH term clusters

**Figure 8:**
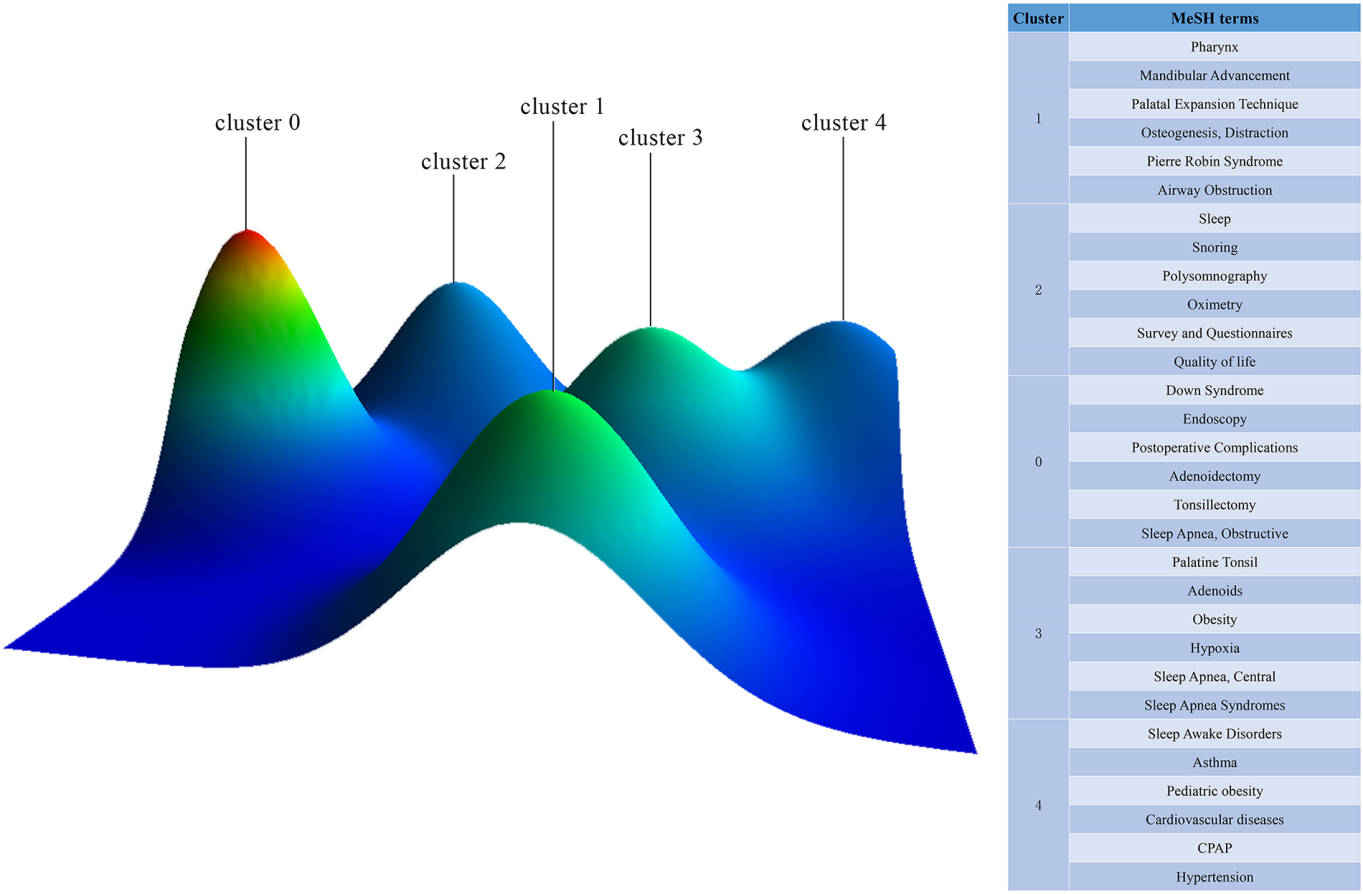
Mountain visualization of the five hot spots by biclustering analysis. The volume of the peaks indicates the quantity of highly frequent Major MeSH terms. A peak's height and color are proportional to its internal similarity and the standard deviation (blue for high deviations and red for low deviations). An interval indicates the degree of similarity between two peaks.

**Figure 9:**
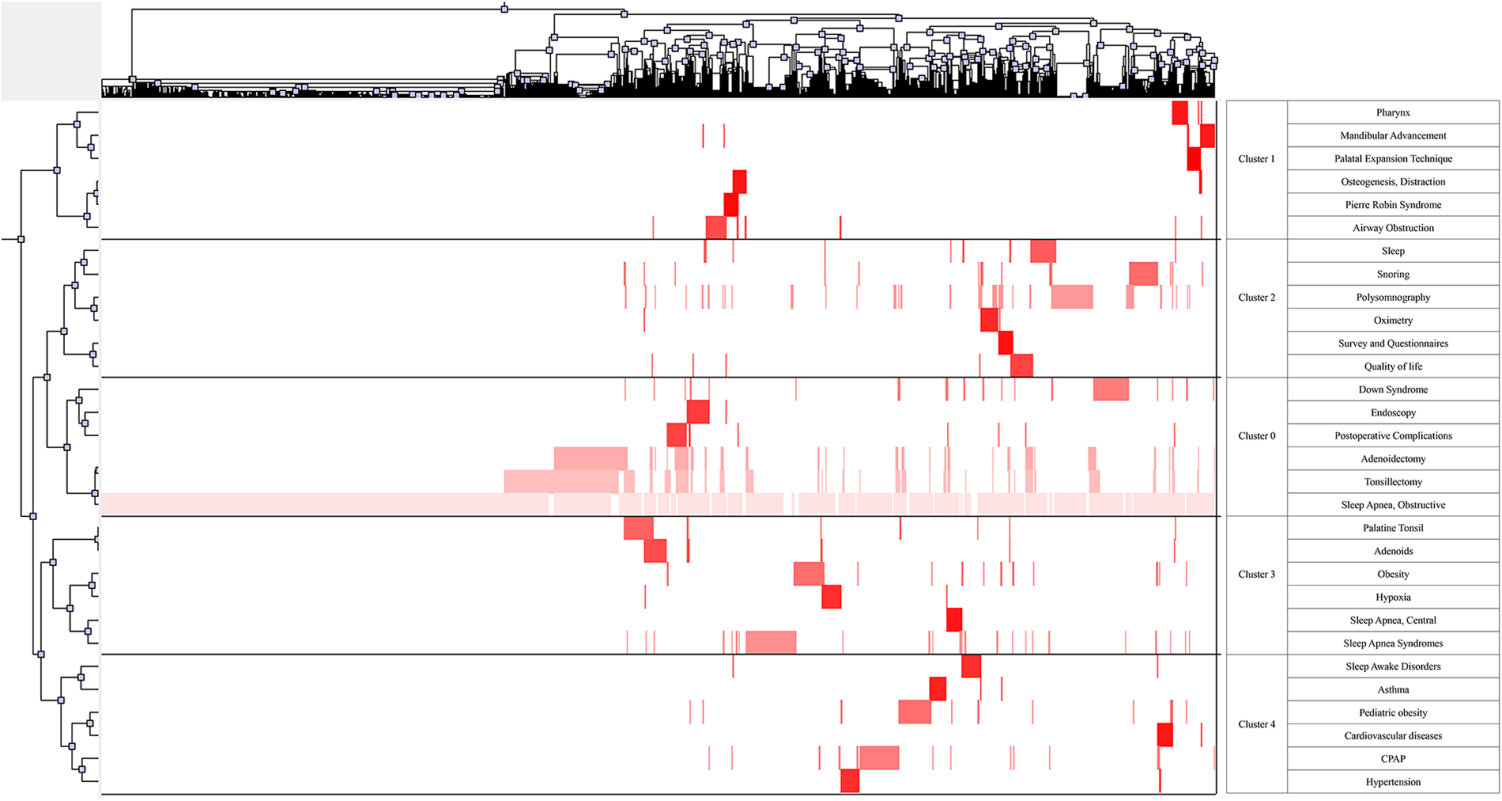
Matrix visualization of the five hot spots by biclustering analysis. Literature is shown at the top, and highly frequent Major MeSH terms are shown on the left. The color of the grid is determined by its frequency (dark red is significant, and white is close to non-significant).

In our study, 1,387 MeSH major topics were retrieved with a cumulative frequency of 8,830. Based on H index evaluation, we defined terms that appear more than 31 times as high-frequent terms. Table 6 summarizes 30 terms extracted from publications that account for 56.74% (5010/8830) of all terms found in publications. Following that, we derived the high-frequency Major MeSH terms 31 rows × 2,627 columns of the co-word matrix. Subsequently, We imported the matrix into the gCluto software for a biclustering analysis. To promote similar row convergence, we reset the rows of the initial matrix and divide each cluster horizontally by a black line. Eventually, The matrix visualization (Figure 8) and mountain visualization (Figure 9) generated by biclustering analysis were divided into 5 clusters, representing different hotspot directions.

**Table 6:** Highly frequent major meSH terms.

Rank	Major MeSH terms	Frequency	Proportion of frequency (%)	Cumulative percentage (%)
1	Sleep Apnea, Obstructive	2,410	27.2,933	27.2,933
2	Tonsillectomy	478	5.4,134	32.7,067
3	Adenoidectomy	307	3.4,768	36.1,835
4	Polysomnography	187	2.1,178	38.3,012
5	Sleep Apnea Syndromes	157	1.778	40.0,793
6	Down Syndrome	119	1.3,477	41.427
7	Continuous Positive Airway Pressure	116	1.3,137	42.7,407
8	Pediatric Obesity	96	1.0,872	43.8,279
9	Obesity	94	1.0,646	44.8,924
10	Snoring	91	1.0,306	45.923
11	Palatine Tonsil	81	0.9,173	46.8,403
12	Sleep	75	0.8,494	47.6,897
13	Adenoids	63	0.7,135	48.4,032
14	Airway Obstruction	62	0.7,022	49.1,053
15	Postoperative Complications	57	0.6,455	49.7,508
16	Quality of Life	56	0.6,342	50.3,851
17	Endoscopy	53	0.6,002	50.9,853
18	Sleep Wake Disorders	46	0.521	51.5,062
19	Hypertension	46	0.521	52.0,272
20	Hypoxia	46	0.521	52.5,481
21	Oximetry	44	0.4,983	53.0,464
22	Asthma	41	0.4,643	53.5,108
23	Mandibular Advancement	38	0.4,304	53.9,411
24	Cardiovascular Diseases	37	0.419	54.3,601
25	Sleep Apnea, Central	37	0.419	54.7,792
26	Pharynx	37	0.419	55.1,982
27	Osteogenesis, Distraction	37	0.419	55.6,172
28	Surveys and Questionnaires	35	0.3,964	56.0,136
29	Pierre Robin Syndrome	33	0.3,737	56.3,873
30	Palatal Expansion Technique	31	0.3,511	56.7,384

OSA-related surgery and postoperative complication (cluster 0); New insights into airway obstruction and new techniques for treatment (cluster 1); Evaluation and diagnosis for childhood OSA (cluster 2); Etiology and pathophysiological mechanisms of OSA (cluster 3); Common complications and comorbidities of OSA (cluster 4).

## 4. Discussion

In recent years, childhood OSA has received increasing attention as more children seek treatment for sleep problems. The statistical and quantitative analysis we conducted revealed that there had been a remarkable improvement in research on childhood OSA from 2013 to 2022, and a growing number of researchers are entering the field. The United States has an absolute leading position in the field of childhood OSA research. Most institutions and authors who have contributed to this field originate in the United States. In comparison with other countries, the United States has advanced equipment, a high-quality experimental environment, and superior clinical trial conditions. In spite of this, countries do not cooperate well among themselves, and research results are not shared efficiently, which impedes global understanding and research efficiency related to childhood OSA. Research on childhood OSA will surely make greater progress if cooperation between countries is strengthened, thereby preventing a great deal of invalid work. Even though there has been a large amount of research on childhood OSA, it remains relatively chaotic and does not include a hotspot analysis. Based on this study, we identified burst keywords in the last decade. Meanwhile, we also interpreted five hot spots on childhood OSA, which are obtained from co-word biclustering. By utilizing these two practical analyses, we could identify hotspots and frontiers in this field.

Cluster 0 focuses on OSA-related surgery and postoperative complication. Adenotonsillectomy (AT) has long been considered the first-line treatment for OSA in children (15). In 2013, The New England Journal of Medicine published a landmark multi-center, randomized controlled trial of adenotonsillectomy (16). This trial compared outcomes in children with OSA who were randomized to watchful waiting or early adenotonsillectomy. After 7months, neurocognitive outcomes were similar in the two groups. However, children in the AT group experienced greater improvements in polysomnography (PSG) parameters, behavior, and quality of life (QoL) scores than those observed. AT has many possible postoperative complications, such as bleeding, hoarseness, difficulty swallowing, and respiratory compromise (17). Children with Down syndrome are at very high risk for OSA, with prevalence rates of 40%–80% compared with prevalence rates of 1%–4% in otherwise healthy children (18). Although AT leads to the successful resolution of OSA in 79% of the general pediatric population, resolution in children with Down syndrome ranges from 6 to 33% (19, 20). Adenotonsillectomy should be considered as a first-line treatment for this population while keeping in mind that monotherapy may not be sufficient in some cases (21). Future studies should further quantify AT outcome indicators and explore other effective adjuvant treatments.

Cluster 1 provides new insights into airway obstruction and techniques for treatment. Pierre Robin Sequence (PRS) is diagnosed by a constellation of characteristics, including micrognathia, glossoptosis, and varying degrees of upper airway obstruction (22, 23). Based on its characteristics, PRS is a leading cause of OSA in newborns. Distraction osteogenesis, like mandibular distraction osteogenesis, is a surgical technique that gradually lengthens the mandible after an osteotomy by using an internal or external distraction device, directly correcting the micrognathia (24). Orthodontists perform rapid palatal expansion on children to reduce nasal resistance, increase nasal volume, elevate tongue posture, and enlarge the pharyngeal airway to treat obstructive sleep apnea (25). Also, mini-implant-assisted rapid palatal expansion has been proven to promote functional breathing (26). Mandibular advancement device (MAD) is a primary treatment alternative for patients with mild to moderate OSA and severe OSA patients who cannot tolerate CPAP (27). By advancing the mandible and tongue towards an anterior position, MAD enlarges the upper airway and reduces its collapsibility while sleeping. Along with technological innovations, an increasing number of alternative treatments for OSA have emerged. Future studies should focus on specific populations, particularly those children with maxillofacial developmental malformations.

Cluster 2 investigates to evaluation and diagnosis of pediatric OSA. PSG parameters, such as AHI, oAHI, PaCO2, and SaO2, have long been considered the gold standard for diagnosing pediatric OSA (28, 29). As a result, doctors and researchers pay more attention to the outcome of PSG, ignoring the real feelings of children. Studies have shown that OSA profoundly affects a child's QoL and behavior (30). Interestingly, the severity of OSA, measured by the obstructive AHI score, does not correlate with the QoL of children with OSA. Consequently, the AHI score does not fully reflect OSA's impact on a child's health and well-being. Thus, it is high time to pay more attention to QoL instruments, such as the OSA-18 questionnaire, to help determine the most appropriate treatment for children with OSA.

Cluster 3 reflects on the etiology and pathophysiological mechanisms of OSA. Traditionally, adenoid and tonsil hypertrophy is considered the most prominent cause of OSA in children. However, several studies have shown that overweight/obese children are more likely to experience the persistence of OSA after an adenotonsillectomy than children within a normal weight range, indicating that obesity is critical in the pathogenesis of OSA (31, 32). There are two main mechanisms through which obesity contributes to OSA. Firstly, the presence of fat at the level of pharyngeal soft tissue reduces lumen caliber and contributes to structural collapse. Secondly, patients have decreased respiratory function due to fat accumulation in the thoracic and abdominal walls (33). Weight-loss management may be critical in treating obesity-related OSA in children, especially for patients who have previously undergone adenotonsillectomy surgery without achieving a satisfactory outcome (34). Despite this, parents do not pay enough attention to their children's weight control (35). Obese-related OSA clinical trials should be expanded, and it is necessary to gather more evidence in the future for this claim.

Cluster 4 focuses on complications and comorbidities of OSA. It has been reported that OSA prevalence can range from 40% to 80% in patients suffering from hypertension, heart failure, coronary artery disease, pulmonary hypertension, atrial fibrillation, and stroke (36). Even though OSA is commonly observed among patients with heart disease, and there is a high risk of adverse cardiovascular outcomes due to OSA-related stressors, OSA is often underrecognized and undertreated in cardiovascular care (37). CPAP should be offered to patients with severe OSA. In this area, future research should focus more on the prevention of OSA-related cardiovascular disease. Asthma and OSA are closely related, which could be attributable to their coexistence, shared risk factors, or distinct interaction mechanisms (38). There should be a better understanding of the relationship between OSA and asthma as soon as possible, both in terms of etiology and therapeutic effects.

## 5. Strength and limitations

Currently, this study represents the first bibliometric analysis of publications specifically focused on childhood OSA, providing directions regarding hotspots and future directions. Despite this, we realized there still has potential limitations to this study. There might be a difference between the bibliographic analysis data and actual research progress because of the rapid increase in related papers on childhood OSA. Further, the computational error of the database may cause a deviation in the result. For example, Web of Science may mislabel document types, resulting in a dataset containing papers that should be filtered out but missing papers that should be included.

## 6. Conclusions

In conclusion, global research over the past ten years has also been fruitful, establishing the foundation for childhood OSA. Our bibliometric visualization analysis reveals childhood OSA research's core power and hotspot evolution. Clusters (0–4) of high-frequency Major Mesh topics have attracted extensive attention. Evaluation and treatment methods of childhood OSA remain major focuses. It is worth noting that numerous clinical trials have focused on a specific group of OSA children, and it should be expanded in the future. We believe this article will provide other researchers with new directions and may contribute to a future breakthrough in this field.

## Data Availability

The original contributions presented in the study are included in the article, further inquiries can be directed to the corresponding authors.

## References

[1] Incerti Parenti S Fiordelli A Bartolucci ML Martina S D'Antò V Alessandri-Bonetti G. Diagnostic accuracy of screening questionnaires for obstructive sleep apnea in children: a systematic review and meta-analysis. Sleep Med Rev. (2021) 57:101464. 10.1016/j.smrv.2021.101464 33827032

[2] Chan KC Au CT Hui LL Wing YK Li AM. Childhood OSA is an independent determinant of blood pressure in adulthood: longitudinal follow-up study. Thorax. (2020) 75(5):422–31. 10.1136/thoraxjnl-2019-213692 32209641

[3] Shen Y Xu Z Shen K. Urinary leukotriene E4, obesity, and adenotonsillar hypertrophy in Chinese children with sleep disordered breathing. Sleep. (2011) 34(8):1135–041. 10.5665/sleep.1178 21804676 PMC3138169

[4] Tseng PH Lee PL Hsu WC Ma Y Lee YC Chiu HM A higher proportion of metabolic syndrome in Chinese subjects with sleep-disordered breathing: a case-control study based on electrocardiogram-derived sleep analysis. PLoS One. (2017) 12(1):e0169394. 10.1371/journal.pone.0169394 28081171 PMC5231382

[5] Kaditis AG Alonso Alvarez ML Boudewyns A Alexopoulos EI Ersu R Joosten K Obstructive sleep disordered breathing in 2- to 18-year-old children: diagnosis and management. Eur Respir J. (2016) 47(1):69–94. 10.1183/13993003.00385-2015 26541535

[6] Cooper ID. Bibliometrics basics. J Med Libr Assoc. (2015) 103(4):217–8. 10.3163/1536-5050.103.4.013 26512226 PMC4613387

[7] Rosas SR Kagan JM Schouten JT Slack PA Trochim WM. Evaluating research and impact: a bibliometric analysis of research by the NIH/NIAID HIV/AIDS clinical trials networks. PLoS One. (2011) 6(3):e17428. 10.1371/journal.pone.0017428 21394198 PMC3048860

[8] Landis JR Koch GG. The measurement of observer agreement for categorical data. Biometrics. (1977) 33(1):159–74. 10.2307/2529310 843571

[9] van Eck NJ Waltman L. Software survey: vOSviewer, a computer program for bibliometric mapping. Scientometrics. (2010) 84(2):523–38. 10.1007/s11192-009-0146-3 20585380 PMC2883932

[10] Synnestvedt MB Chen C Holmes JH. Citespace II: visualization and knowledge discovery in bibliographic databases. AMIA Annu Symp Proc. (2005) 2005:724–8. 16779135 PMC1560567

[11] Online Analysis Platform: BIBLIOMETRC. 2013. Available at: http://bibliometric.com

[12] Lei C WL Lei Y Han Z Yuefang H Yingna H Hao Z. Development of a text mining system based on the co-occurrence of bibliographic items in literature databases. New Technol of Library and Infor Ser. (2008) 8:70–5. 10.3969/j.issn.1003-3513.2008.08.013

[13] Lab K. gCLUTO-Graphical Clustering Toolkit. 2014. Available at: http://glaros.dtc.umn.edu/gkhome/cluto/gcluto/download

[14] Hao KJ Jia X Dai WT Huo ZM Zhang HQ Liu JW Mapping intellectual structures and research hotspots of triple negative breast cancer: a bibliometric analysis. Front Oncol. (2021) 11:689553. 10.3389/fonc.2021.689553 35047380 PMC8763010

[15] Marcus CL Brooks LJ Draper KA Gozal D Halbower AC Jones J Diagnosis and management of childhood obstructive sleep apnea syndrome. Pediatrics. (2012) 130(3):576–84. 10.1542/peds.2012-1671 22926173

[16] Marcus CL Moore RH Rosen CL Giordani B Garetz SL Taylor HG A randomized trial of adenotonsillectomy for childhood sleep apnea. N Engl J Med. (2013) 368(25):2366–76. 10.1056/NEJMoa1215881 23692173 PMC3756808

[17] De Luca Canto G Pachêco-Pereira C Aydinoz S Bhattacharjee R Tan HL Kheirandish-Gozal L Adenotonsillectomy complications: a meta-analysis. Pediatrics. (2015) 136(4):702–18. 10.1542/peds.2015-1283 26391937 PMC9923592

[18] Marcus CL Keens TG Bautista DB von Pechmann WS Ward SL. Obstructive sleep apnea in children with down syndrome. Pediatrics. (1991) 88(1):132–9. 10.1542/peds.88.1.132 1829151

[19] Maris M Verhulst S Wojciechowski M Van de Heyning P Boudewyns A. Outcome of adenotonsillectomy in children with down syndrome and obstructive sleep apnoea. Arch Dis Child. (2017) 102(4):331–6. 10.1136/archdischild-2015-310351 27484971

[20] Nation J Brigger M. The efficacy of adenotonsillectomy for obstructive sleep apnea in children with down syndrome: a systematic review. Otolaryngol Head Neck Surg. (2017) 157(3):401–8. 10.1177/0194599817703921 28485249

[21] Seither K Helm BM Heubi C Swarr D Suhrie KR. Sleep apnea in children with down syndrome. Pediatrics. (2023) 151(3):e2022058771. 10.1542/peds.2022-058771 36762410

[22] Robin P. A fall of the base of the tongue considered as a new cause of nasopharyngeal respiratory impairment: pierre robin sequence, a translation. 1923. Plast Reconstr Surg. (1994) 93(6):1301–3. 10.1097/00006534-199405000-00032 8171154

[23] Hsieh ST Woo AS. Pierre robin sequence. Clin Plast Surg. (2019) 46(2):249–59. 10.1016/j.cps.2018.11.010 30851756

[24] Breugem CC Logjes RJH Nolte JW Flores RL. Advantages and disadvantages of mandibular distraction in robin sequence. Semin Fetal Neonatal Med. (2021) 26(6):101283. 10.1016/j.siny.2021.101283 34663561

[25] Fernández-Barriales M Lafuente-Ibáñez de Mendoza I Alonso-Fernández Pacheco JJ Aguirre-Urizar JM. Rapid maxillary expansion versus watchful waiting in pediatric OSA: a systematic review. Sleep Med Rev. (2022) 62:101609. 10.1016/j.smrv.2022.101609 35286895

[26] Brunetto DP Moschik CE Dominguez-Mompell R Jaria E Sant'Anna EF Moon W. Mini-implant assisted rapid palatal expansion (MARPE) effects on adult obstructive sleep apnea (OSA) and quality of life: a multi-center prospective controlled trial. Prog Orthod. (2022) 23(1):3. 10.1186/s40510-021-00397-x 35102477 PMC8804045

[27] Uniken Venema JAM Rosenmöller B de Vries N de Lange J Aarab G Lobbezoo F Mandibular advancement device design: a systematic review on outcomes in obstructive sleep apnea treatment. Sleep Med Rev. (2021) 60:101557. 10.1016/j.smrv.2021.101557 34662769

[28] Sateia MJ. International classification of sleep disorders-third edition: highlights and modifications. Chest. (2014) 146(5):1387–94. 10.1378/chest.14-0970 25367475

[29] [Chinese guideline for the diagnosis and treatment of childhood obstructive sleep apnea (2020)]. Zhonghua Er Bi Yan Hou Tou Jing Wai Ke Za Zhi. (2020) 55(8):729–47. 10.3760/cma.j.cn115330-20200521-00431 32791771

[30] Baldassari CM Mitchell RB Schubert C Rudnick EF. Pediatric obstructive sleep apnea and quality of life: a meta-analysis. Otolaryngol Head Neck Surg. (2008) 138(3):265–73. 10.1016/j.otohns.2007.11.003 18312869

[31] Mitchell RB Kelly J. Outcome of adenotonsillectomy for obstructive sleep apnea in obese and normal-weight children. Otolaryngol Head Neck Surg. (2007) 137(1):43–8. 10.1016/j.otohns.2007.03.028 17599563

[32] Andersen IG Holm JC Homøe P. Obstructive sleep apnea in children and adolescents with and without obesity. Eur Arch Otorhinolaryngol. (2019) 276(3):871–8. 10.1007/s00405-019-05290-2 30689039

[33] Fiorino EK Brooks LJ. Obesity and respiratory diseases in childhood. Clin Chest Med. (2009 Sep) 30(3):601–8. x. 10.1016/j.ccm.2009.05.010 19700055

[34] Ersu R Chen ML Ehsan Z Ishman SL Redline S Narang I. Persistent obstructive sleep apnoea in children: treatment options and management considerations. Lancet Respir Med. (2023) 11(3):283–96. 10.1016/s2213-2600(22)00262-4 36162413

[35] Andersen IG Holm JC Homøe P. Impact of weight-loss management on children and adolescents with obesity and obstructive sleep apnea. Int J Pediatr Otorhinolaryngol. (2019) 123:57–62. 10.1016/j.ijporl.2019.04.031 31075707

[36] Yeghiazarians Y Jneid H Tietjens JR Redline S Brown DL El-Sherif N Obstructive sleep apnea and cardiovascular disease: a scientific statement from the American heart association. Circulation. (2021) 144(3):e56–67. 10.1161/cir.0000000000000988 34148375

[37] Kwok KL Ng DK Chan CH. Cardiovascular changes in children with snoring and obstructive sleep apnoea. Ann Acad Med Singap. (2008) 37(8):715–21. 10.47102/annals-acadmedsg.V37N8p715 18797568

[38] Prasad B Nyenhuis SM Imayama I Siddiqi A Teodorescu M. Asthma and obstructive sleep apnea overlap: what has the evidence taught US? Am J Respir Crit Care Med. (2020) 201(11):1345–57. 10.1164/rccm.201810-1838TR 31841642 PMC7258643

